# Management of Stable Vitiligo—A Review of the Surgical Approach

**DOI:** 10.3390/jcm12051984

**Published:** 2023-03-02

**Authors:** Małgorzata Grochocka, Adam Wełniak, Aleksandra Białczyk, Luiza Marek-Jozefowicz, Tadeusz Tadrowski, Rafał Czajkowski

**Affiliations:** 1 Department of Dermatology and Venerology, Faculty of Medicine, Collegium Medicum in Bydgoszcz, Nicolaus Copernicus University in Toruń, 85-094 Bydgoszcz, Poland; 2 Student Research Group of Experimental Dermatology, Department of Dermatology and Venerology, Faculty of Medicine, Collegium Medicum in Bydgoszcz, Nicolaus Copernicus University in Toruń, 85-094 Bydgoszcz, Poland; 3 Student Research Group of Dermatology, Department of Dermatology and Venerology, Faculty of Medicine, Collegium Medicum in Bydgoszcz, Nicolaus Copernicus University in Toruń, 85-094 Bydgoszcz, Poland

**Keywords:** stable vitiligo, repigmentation, therapy tissue grafts, cellular grafts, surgical treatment

## Abstract

At present, vitiligo is the most common depigmenting skin disorder, characterized by clearly demarcated discolored patches of various shapes and sizes. Depigmentation results from the initial dysfunction and subsequent destruction of melanin-producing cells, called melanocytes, which are located in the basal layer of the epidermis and in hair follicles. This review concludes that the extent of repigmentation, regardless of the treatment method, is greatest in stable localized vitiligo patients. The aim of this review is to provide an overview of the clinical evidence for which the vitiligo treatment method (cellular or tissue) is more effective. The treatment relies on multiple factors, ranging from patient skin predisposition for repigmentation to the experience of the facility performing the procedure. Vitiligo is a significant problem in modern society. Although it is a typically asymptomatic and not life-threatening disease, it may have significant psychological and emotional impacts. Standard treatment relies on pharmacotherapy and phototherapy; however, the treatment of patients with stable vitiligo varies. The stability of vitiligo more than often implies the exhaustion of the potential for skin self-repigmentation. Thus, the surgical methods that distribute normal melanocytes into the skin are crucial elements of these patients’ therapy. The most commonly used methods are described in the literature, with an indication of their recent progress and changes. In addition, information on the efficiency of the individual methods at specific locations is compiled in this study, and the prognostic factors indicating repigmentation are presented. Cellular methods are the best therapeutic option for large-sized lesions; although they are more exorbitant than tissue methods, they benefit from more rapid healing times and presenting fewer side effects. Dermoscopy is a valuable tool used to assess the further course of repigmentation, where it is of great value to evaluate the patient prior to and following an operation.

## 1. Introduction

Vitiligo is a common depigmenting skin disorder affecting approximately 0.5–2% of the population, worldwide, for both sexes and all races [1], characterized by well-demarcated white patches resulting from the selective loss of melanocytes [2]. The lesions are most often localized on the face, dorsal aspects of the hands, feet, nipples, axilla, sacral, inguinal, umbilical and anal areas [3]. The dynamics, clinical course and extent of the disease process sometimes widely vary. In the initial stage, the number of white spots is usually low, and their size varies from a few mm to a few cm [4]. Vitiligo is a multifactorial disease with a complex etiology. Numerous hypotheses have been proposed in the literature in an attempt to explain the mechanisms leading to melanocytes’ dysfunction and destruction. These include genetic, autocytotoxic (oxidative stress), autoimmune, autoinflammatory, neuronal (neural), apoptotic and adhesion disorder mechanisms. None of the proposed hypotheses fully explain the phenotypic diversity of vitiligo; therefore, the multifactorial theory is, to date, the most valid [5,6,7]. The most popular theory of vitiligo development is a multifactorial hypothesis, according to which genetic conditions predispose vitiligo macules to occur due to specific environmental factors [2,8]. Vitiligo is manifested in genetically predisposed individuals, following the action of exo- and/or endogenous factors that induce cellular stress, initiating autoimmune and autoinflammatory processes, destroying pigment cells [5]. Vitiligo is inherited in a polygenic manner. Multiple alleles at multiple unlinked gene loci are involved in the etiopathogenesis of disease. Scientific reports indicate that in approximately 20% of patients, at least one first-degree relative is also affected by the condition [9]. Genome-wide association studies (GWASs) involve genotyping a set of highly abundant single-nucleotide polymorphisms (SNPs) across the genome to demonstrate the most common genetic variation associated with a disease or to identify quantitative heritable traits that are risk factors for disease development [10]. Five GWASs have been performed for vitiligo, and more than 50 loci associated with the risk of disease were identified [6]. Through GWASs, susceptibility genes have been discovered that are responsible for controlling pigment cell homeostasis. They are mainly related to melanin biosynthesis, the antioxidant system and regulation of the immune system. Vitiligo susceptibility genes identified from GWASs include NALP-1, CTLA4, TGFBR2, PTPN22, LPP, IL-2RA, UBASH3A and RERE [11]. GWASs have identified many genes associated with vitiligo, including genes crucial for antigen presentation (e.g., MHC classes I and II); T-cell development, e.g., CD44; T-cell receptor signaling; T-cell activation, e.g., CD80; melanocyte homeostasis and melanogenesis; e.g., TYR; MC1R and apoptosis. Vitiligo also often presents as an autoimmune disease due to the presence of melanocyte-specific antibodies and its association with other autoimmune diseases, such as alopecia areata, Hashimoto’s thyroiditis, pernicious anemia, type 1 diabetes and systemic lupus erythematosus [12,13]. The autoinflammatory theory posits that melanocytes induce a non-specific immune response by secreting exosomes that contain melanocyte-specific antigens: microRNAs (miRNAs), heat shock protein 70 (Hsp70) and proteins that act as damage-associated molecular patterns (DAMPs). Exosomes deliver target antigens associated with vitiligo to nearby dendritic cells, inducing their maturation and antigen presentation to T lymphocytes, thus linking cellular stress and acquired immunity [2]. As noted in several studies, there is extensive evidence that oxidative stress plays a significant role in promoting the onset of vitiligo. Reactive oxygen species (ROS), induced by multiple intrinsic factors, oxidize components of cells, leading to melanocyte destruction and creating depigmented macules [14]. High oxidative stress markers, such as hydrogen peroxide, superoxide dismutase and malondialdehyde, and reduced protective mechanisms against these oxidative stressors, such as catalase and glutathione peroxidase, were documented in the skin and blood of vitiligo patients [15]. Defects in the number and function of regulatory T cells may also contribute to the pathogenesis of vitiligo. Melanocyte-specific, cytotoxic CD8+ T cells have been greatly involved in melanocyte destruction. Patients with vitiligo had more cytotoxic CD8+ T cells in the blood compared to healthy subjects [14,16].

The choice of therapeutic method depends on a number of factors, including the type of vitiligo, the distribution of skin lesions, as well as the patient’s age and skin phototype. Studies show that the prevalence of depression in vitiligo patients is high, and vitiligo can significantly affect their quality of life. There are many surgical and non-surgical therapeutic approaches used to treat vitiligo at present; however, none can guarantee the complete and long-lasting disappearance of the disease [17]. The Vitiligo Subcommittee of the European Dermatology Forum, in 2012, presented guidelines for the management and treatment of vitiligo [18]. Therefore, discovering an effective therapy for the patient is significant but remains challenging. Treatments range from phototherapy (NB-UVB 311 nm, PUVA), topical corticosteroids and immunomodulators, systemic corticosteroids [19] and vitamin D analog (e.g., calcipotriol) [20] antioxidants [21] to surgical techniques [22], combined methods and also camouflage or depigmentation procedures [18]. A greater response to treatment observed seen in younger patients, disease of recent onset, darker skin types and in lesions on the face, neck and trunk [23]. For the management of vitiligo, several guidelines have been released in the field of study [18,23,24,25]. Topical corticosteroids (TCs) administered once daily are a standard first-line and adjunctive therapy [18,24]. TCs work best on sun-exposed areas, such as the face and neck, for dark skin, and in a recent lesion [23,24]. In contrast, systemic corticosteroids are a choice for active, rapidly progressive lesions. For limited forms of vitiligo, especially the periocular region of the face, topical calcineurin inhibitors can be used as an alternative to TCs. UV-light therapy is the first-line treatment for individuals who have an inadequate response to topical therapy or have an extensive disease. Additional therapy (topical calcineurin inhibitor or TCs) is associated with better outcomes [24]. At present, NB-UVB represents the preferred choice of active and generalized vitiligo phototherapy [18]. Camouflage techniques are a significant element of disease therapy, too, especially considering how the disease affects the patient’s perception of their own body [25].

To date, topical Janus kinase inhibitors (JAKs), which regulate the immune response by blocking the IFN-CXCL10 axis [26], are being investigated as a potential treatment option for vitiligo [27]. Preliminary reports suggest they are more effective than topical calcineurin inhibitors and at least equal to topical corticosteroids [28]. Ruxolitinib, FDA-approved for the treatment of myelofibrosis and polycythemia, administered orally, achieved the rapid remission of lesions on the face and torso in vitiligo patients. Ruxolitinib also caused a number of side effects, including thrombocytopenia, anemia and neutropenia [29]. However, no studies support their effectiveness in treating stable vitiligo, and their use is severely limited due to the side effects observed in patients. In 2022, the United States Food and Drug Administration (FDA) approved ruxolitinib cream for the treatment of non-segmental vitiligo in adults. The first FDA-approved ruxolitinib cream for the treatment of nonsegmental vitiligo in adult and pediatric patients that were 12 years of age and older [30].

There are very high hopes for the use of the hormone L-MSH, which protects melanocytes from apoptosis induced by oxidative stress in the treatment of vitiligo. Its action is to reduce the production of hydrogen peroxide in response to UV exposure. L-MSH, through the activation of the AKT pathway, increases levels of the transcription factor MITF, which regulates the expression of numerous enzyme protein genes, such as catalase [31].

Surgical methods are the most crucial therapeutic tool for patients with stable vitiligo because they can be used in cases of vitiligo resistant to other therapies [32,33]. Determining whether the disease is stable is mainly necessary in the context of making appropriate therapeutic decisions. The stability of the disease is the primary criterion for referring a patient for surgical treatment. Stability is described as an absence of new lesions, a lack of enlargement in the size of pre-existing macules and the nonappearance of Koebner’s phenomenon over a certain period of time [21]. To date, there is no consensus on a time period of stability, and the span varies from 4 months to 3 years, according to different authors [16,34,35]. Additionally, it is still debated whether global disease stability or lesional stability is more important [36]. As for disease inactivity, segmental vitiligo usually becomes inactive after the disease has been stable for 2 years; however, non-segmental vitiligo is prone to reactivation, even after prolonged periods of stability [34]. The histological parameters observed in active vitiligo are spongiosis, epidermal lymphocytes and basal-layer vacuolization in the epidermis and dermal lymphocytes, along with melanophages in the dermis [35]. The lesions are dominated by a population of CD4+ and CD8+ T cells, with a reduced number of natural regulator T cells [37]. The Vitiligo Signs of Activity Score, evaluating the appearance of confetti-like depigmentation, Koebner phenomenon and hypochromic areas/borders, appears to be a valid and reliable instrument to assess disease stability [38]. Laboratory markers, such as CXCL9 or S100B protein, might be linked to vitiligo activity and display promising results as potential diagnostic tools of disease stability [39,40,41]. Disease stability is best assessed using a combination of clinical assessment criteria (VASI or VETF), patient self-assessment and digital imaging of individual skin lesions over the past 12 months [4].

The surgical approach to vitiligo treatment can be divided into two branches: tissue and cellular grafting. Suction blister epidermal grafting (SBEG) and mini-punch grafting (MPG) are most prevalent in tissue grafting and epidermal cell suspension (ECS) and follicular cell suspension (FCS) in cellular grafting [20,22,42].

The present review aims to evaluate the most commonly used surgical treatment methods for vitiligo with regard to their procedure, the advantages and disadvantages of each method and to determine which patients will benefit most from which method. Based on the reviewed papers, we conclude that these methods are the most commonly used in the field, which is why we decided to describe them in detail regarding their procedure, the advantages and disadvantages of each method and to determine which patients will benefit most from which method.

## 2. Materials and Methods

We conducted a systematic review according to PRISMA (Preferred Reporting Items for Systematic Review and Meta-Analyses) criteria. The guidelines are primarily intended to aid in the conduct of systematic reviews; however, they can also be useful in the context of narrative reviews. We used the PRISMA guidelines, which ensured that the narrative review was comprehensive, transparent and well-structured. We used PRISMA guidelines to only track and collect the articles, which we used to conduct a narrative review.

The articles evaluated were those identified by searching PubMed and ClinicalKey. We searched for articles using the following keywords: vitiligo, surgical treatment and stable vitiligo. Our search was restricted to the English language. One of the filters used was the initial limitation of the searched publications to the last 5 years; however, during the writing process, older articles were also included in the analysis. The search was conducted from December 2021 to November 2022. In total, 250 articles were obtained, and 80 of them were analyzed.

## 3. Results

**Figure 1:**
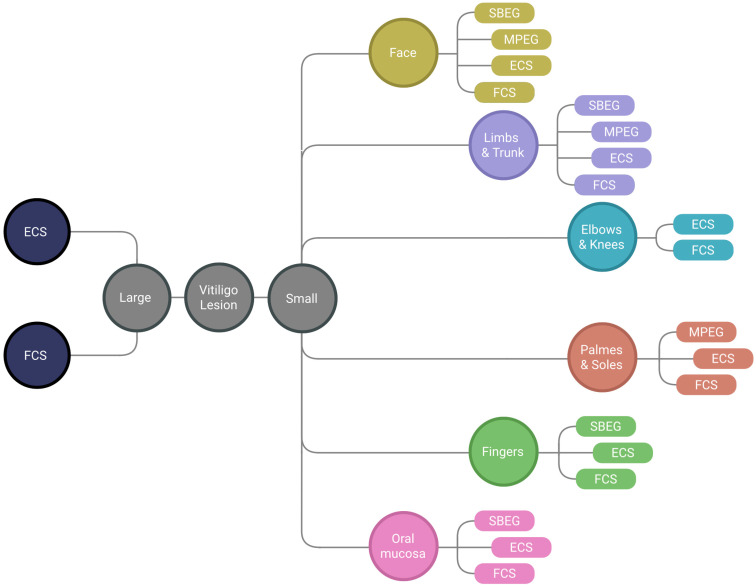
Method selection based on lesion location. SBEG—suction blister epidermal grafting; MPG—mini-punch grafting; ECS—epidermal cell suspension; FCS—follicular cell suspension.

Only the most commonly used methods were described, thus excluding others, such as split-thickness skin graft, epidermal curettage technique, smash graft, flip-top pigment grafting and hair follicle graft, since they are less frequently performed [22,43]. Slightly better results have been reported for cultured variants of the ECS and FCS methods. However, the costs associated with these practices are disproportionate to the effects. Hence, cultured methods have not found relevance in the field. This is why tissue methods are referred to as epidermal/follicular cell suspension instead of noncultured epidermal/follicular cell suspension. Every method has its advantages and limitations, and each can be applied to any body part. Some offer significantly better results than others when applied to a specific area. A summary of the choice of treatment method is presented in Figure 1.

Other factors, such as the presence of scars, are associated with poor response and increased risk of complications [44]. In addition to method selection, patient compliance and follow-up treatment are very important to reach optimal repigmentation. The tissue graft method involves obtaining a piece of healthy skin from an area of the body unaffected by vitiligo and transferring it to depigmented skin. SBEG uses negative pressure to create blisters applied and fixed to the recipient site. The grafts result in color matching and good cosmetic outcomes [45]. MPG involves creating punctures 1.5 or 1.2 mm in diameter on the depigmented lesion, followed by transference to the donor site, with pigmented skin obtained by punch biopsy from the healthy recipient site. This method is established as the fastest, which, over the years, has been improved, and the maximum increase in pigment spread achieved in tests has increased from 1 to 12 mm [46]. Tissue methods rely on the centrifugal spread of melanocytes from grafted tissue to the diseased skin. New alterations are being sought in the field of study that try to improve them [47,48]. An issue concerning SBEG and MPG is the difficulty in maintaining the graft on uneven surfaces, such as the knees and elbows, and highly mobile areas, such as the lips [45].

Cellular techniques involve obtaining a split-thickness skin graft, separating the epidermis from the skin and creating a suspension from the acquired epidermis. The prepared suspension is spread on the dermabrazed recipient site [43]. The keratinocytes in the suspension help deliver factors that sustain and promote melanocyte growth, resulting in better repigmentation results [48]. The cellular method relies on a diffuse-spread melanocyte pattern. These methods achieve excellent results but are limited by cost and equipment requirements [49]. ECS involves using melanocytes obtained from the epidermis, while FCS exploits melanocytes obtained from the hair follicles. To date, both methods produce similar results; however, the FCS may become the best method with improvements in aspects, such as tissue harvesting and cell preparation [50].

### 3.1. Tissue Grafting Methods

#### 3.1.1. Suction Blister Epidermal Grafting (SBEG)

SBEG was first described by Falabella in 1971 [51] and is widely used for vitiligo treatment in China [52]. The procedure is initiated by preparing the blisters. Suction blisters are raised from the donor site (the medial aspect of the arm, thigh, abdomen or back) using negative pressure from suction plates, cups or inverted syringes with the plunger removed and attached to an automated or manual suction device [53,54]. One of the simplest ways to produce blisters on donor sites is using a cannula and 20 or 50 mL syringes, which can generate the negative pressure required to create blisters [55]. Other options include using an angiosterrometer, a modified gastric suction pump or a double-syringe device [56]. Depending on the age of the patients and donor site, after 30 to 180 min, small vesicles are formed, changing over time into blisters [57]. The small-diameter syringes or cups and preheating of the donor area shorten the suction blister induction time, while an intra-blister saline injection increases the blister size and turns the multilocular blisters into unilocular ones [58]. The dermal injection of lidocaine or saline can hasten blister induction. The recipient site is then cleaned, anesthetized and dermabrazed until pinpoint bleeding is visible, indicating that the dermo-epidermal junction has been reached. The roofs of the blisters at the donor site are removed and transferred to the prepared recipient site. The bandage should be applied to the recipient site for one week, and a judicious use of antibiotic ointment at both sites is recommended. After about one to two weeks, the grafts will detach, leaving behind the transferred melanocytes [59]. Complementing SBEG therapy with concomitant PUVA or narrow-band ultraviolet B (NB-UVB) phototherapy is necessary [59,60]. SBEG is considered a low-cost and effective procedure that can even be performed as a bedside procedure in patients unsuitable for more extensive interventions [61,62]. It can be used around the sensitive areas of the mouth and eyelids [61]. However, despite the low cost and lack of special equipment, SBEG is a time-consuming practice for doctors. For patients, SBEG is an efficient method but may lead to discomfort and slight pain. It must be stated that this method is unsuitable for treating larger areas, uneven surfaces or palms [53]. Undesirable effects of the treatment in the recipient area include hyperpigmentation, per graft halo, infection, color mismatch, reactivation or progression on the recipient site and the Koebner phenomenon, along with hyperpigmentation at the donor site [62,63].

#### 3.1.2. Mini-Punch Grafting (MPG)

**Figure 2:**
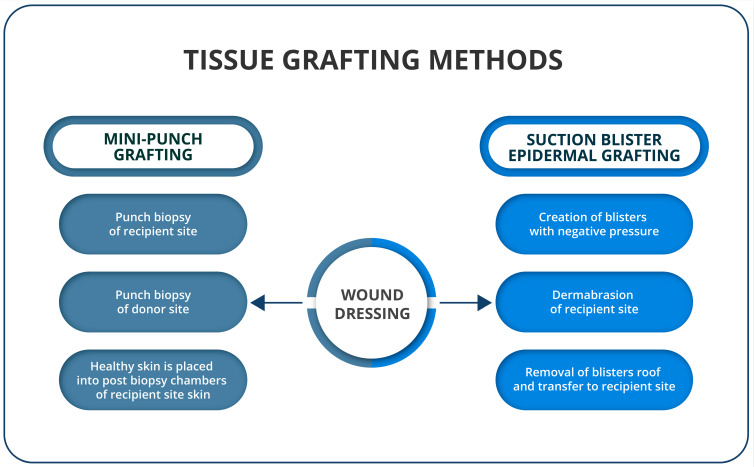
Course of tissue grafting methods.

MPG, as well as SBEG, is an easy-to-perform, rapid and affordable surgical method (Figure 2). A punch biopsy of 1.2–1.5 mm in size and 0.5–3 mm in depth is performed from the normally pigmented donor site, mainly the lateral or inner thigh or gluteal region [59]. The recipient site is prepared using a punch biopsy tool to create chambers that are placed approximately 5 mm apart [64]. Healthy skin obtained from the grafting site is placed in the chambers following biopsy and pressed with gauze until the bleeding stops. Note that the procedure can be completed using tissue glue. Finally, a dressing is applied. Some use splints to immobilize the surgical site [65]. Repigmentation is observed approximately 2 to 3 weeks postoperatively, and individual spots should coalesce between 4 and 6 months [64] Kovacs et al. suggested that, in response to MPG and the following therapy, melanocytes start to horizontally migrate and repopulate the depigmented areas, thanks to the down-modulation of adhesion molecules and the induction of mitogenic mediators [66]. Postoperative treatment with NB-UVB has also been shown to help in the repigmentation process of the lesions [67,68,69]. MPG requires no special equipment or additional personnel and can be easily performed in an outpatient setting [63]. It is suitable for challenging locations, such as the lips, palms, soles and fingers. Nonetheless, it is a time-consuming method, which is not suitable for body folds, oral commissures or non-keratinized mucosa. Side effects occurring at the donor site might include textural and pigmentary variations, such as polka dots, color-mismatch hyperpigmentation and cobblestoning, which is the most common complication of MPG [55,63]. Cobblestoning is a raised-skin, hypertrophic scar, resembling cobblestones. A larger biopunch size is associated with better repigmentation results but at the cost of a higher risk of producing cobblestoning [55]. When this method is applied to larger lesions, serial procedures might be required to be performed at three- to four-week intervals. Depending on repigmentation expansion, additional grafts may be necessary. Classical MPG can be modified by the use of intradermal injections of 0.1 mL platelet-rich plasma (PRP) into graft sites. Such an addition shows better repigmentation compared to the classical method when paired with additional phototherapy combinations during the first 8 weeks of the study. However, this effect was no longer statistically significant when 20 weeks had passed following treatment [65]. The addition of PRP injection is worth considering, as it is not associated with any side effects and significantly increased levels of bFGF in the grafted skin, leading to the migration and proliferation of melanocytes. Furthermore, silicone gel must be observed as a possibility for use in the procedure since it improves the stratum corneum’s hydration and facilitates the regulation of collagen production by fibroblasts [70]. It may also protect against bacteria and stimulate collagen synthesis and the modulation of growth factor expression to restore the balance of fibrogenesis and fibrolysis. During clinical trials, silicone gel applied to the treated lesions was associated with improvements in repigmentation but did not reduce the risk of cobblestoning [71]. Likewise, weekly transverse needling sessions can increase MPG efficiency. Ragab et al. showed that this method hastened and improved the repigmentation extent of stable, resistant, non-segmental vitiligo lesions [72].

### 3.2. Cellular Grafting Methods

#### 3.2.1. Epidermal Cell Suspension (ECS)

ECS, also known as noncultured epidermal cell suspension, as a method, dates back to 1992 [73]. Since then, ECS has been modified to achieve an optimal technique. Modifications include different tissue harvesting methods, the trypsinization method, the medium used for suspending cells, recipient site preparation, dressings used or additional treatment following surgery. The normally pigmented skin of approximately 3–5 cm² is harvested from the lateral thigh or gluteal area. It is noteworthy that the same area can be used for more than one graft without the risk of lower repigmentation [74]. The grafting site is cleaned using povidone–iodine lotion and then anesthetized with 2% lignocaine. The ultrathin skin graft is obtained with a silver skin-grafting knife or a razor blade attached to artery forceps [75]. The donor-to-recipient ratio varies, but is usually 1:10 [76,77], although some use different ratios (1:5 [78] or 1:3 [76]). The use of a lower donor-to-recipient ratio transfers into a lower expansion ratio, leading to better repigmentation, with a higher average percentage improvement in the Vitiligo Area Severity Index (VASI), as well as better color-matching [76,79]. Then, the skin is placed in a saline solution. A good-quality graft floats in the saline without curling at the sides [80]. The relocation of the skin graft into phosphate-buffered saline (PBS) allows for the removal of contaminated fat and blood [74]. The harvested skin is then fragmented into smaller pieces and placed in 4–10 mL of 0.25% trypsin 0.05% ethylenediaminetetraacetic (EDTA) solution, in which the skin is incubated for 40–50 min at 37 °C [75,76,77,81], or even 1.5 h at 39 °C [82]. Warm trypsinization is the most commonly used technique due to its superiority in comparison to cold trypsinization and significantly better time efficiency than room-temperature trypsinization [83]. Forceps are used to separate the epidermis from the dermis; then, the dermis is discarded and the epidermis is broken into small pieces. The suspension is washed in PBS and centrifuged for 4 min at 3200 [56] or 1000 rpm for 5 min [81,83] or, less commonly, for 10 min at a 2000 rpm speed [75]. A pellet containing cells obtained from the basal layer rich in melanocytes is removed from the centrifugation. The prepared sediment is placed into a syringe with PBS, and the suspension is ready for injection. Viability is assessed by light microscopy after staining with trypan blue [55,76]. Additionally, the number of cells can be measured using a hemocytometer [76]. The recipient’s skin at the lesion site is dermabrazed with a motorized dermabrader under local anesthesia until pinpoint bleeding is noted, indicating that a dermo-epidermal junction has been reached. It is best to extend 3 mm beyond the borders of the lesion to avoid a halo of depigmentation [75]. If any bleeding occurs, the area should be washed with a saline solution, and the procedure should be resumed when the bleeding stops. The dermabrasion can be performed using Er:YAG or CO_2_ lasers [83] and allows for a bloodless field during the procedure, although using mechanical dermabrasion is advised. Mechanical dermabrasion may cause more bleeding to occur during ablation; however, it is associated with less thermal damage and the induction of growth factor release, which is beneficial for repigmentation [77]. The cell suspension is then evenly injected over the recipient area with a needle or pipette. The graft site is covered with a meshed collagen sheet, promoting cellular growth and vascularization. Finally, a secondary dressing of sterile gauze is applied. The patient is advised to refrain from moving the treated areas for 30 min, especially if the operating area is located on a limb [75]. Preoperative antibiotics are not usually prescribed to the patient [84]; nonetheless, some medics recommend using anti-inflammatory medication and antibiotics for one week [76]. After one week, the dressing is removed, and the patient returns to the clinic to assess the progress of repigmentation. Follow-up visits are recommended for up to six months since most of the effects should be visible by that time, although repigmentation can persist for as long as 72 months [84]. The photographic record is of great value, allowing for an objective evaluation. Two weeks after the procedure, an NB-UVB [84] treatment can be applied to stimulate repigmentation [75]. It is worth noting that phototherapy should be included only after the procedure has been performed. The usage of NB-UVB prior to treatment does not influence repigmentation following the ECS procedure [85]. Topical steroids or topical calcineurin inhibitors can be applied as an alternative to phototherapy [84]. Factors, such as vitiligo subtypes, the anatomic region affected/treated, as well as family history, need to be taken into account since they are key indicators of the possibility of relapse (Table 1) [77,85]. Formerly known as difficult-to-treat areas, the elbow, knee, acral, eyelid and mucosa show no statistical differences in repigmentation compared to non-complex areas treated when both were treated with ECS [85]. The ECS procedure is fairly well-known in the field and can be performed within one day, and it has a remarkable effect when applied to treat large lesions. Undesirable side effects of the treatment that patients should be aware of are hyperpigmentation [55], infections [81], a halo surrounding the graft [83] and skin atrophy [42]. Notably, hyperpigmentation is usually presented in skin phototypes IV–VI and hypopigmentation and erythema occur in skin phototypes I–III [84]. One of the flaws of the standard technique is an uneven distribution of cell suspension when the treatment concerns an anatomical site with a curve or slope. To prevent this, the basic method can be altered by mixing hyaluronic acid with a cell solution for injection, resulting in a gel-like liquid. This form prevents the leakage and wastage of the solution, and it allows for a more even distribution of the cells [86]. In studies, the addition of growth factors, crucial for melanocyte development, known as basic fibroblast growth factor (bFGF) and cyclic adenosine monophosphate (cAMP), at values of 5 ng/mL recombinant bFGF and 25 mg/500 mL cAMP, was associated with better repigmentation results. However, it should be acknowledged that such a modification of the technique is associated with increased costs [87].

#### 3.2.2. Follicular Cell Suspension (FCS)

**Figure 3:**
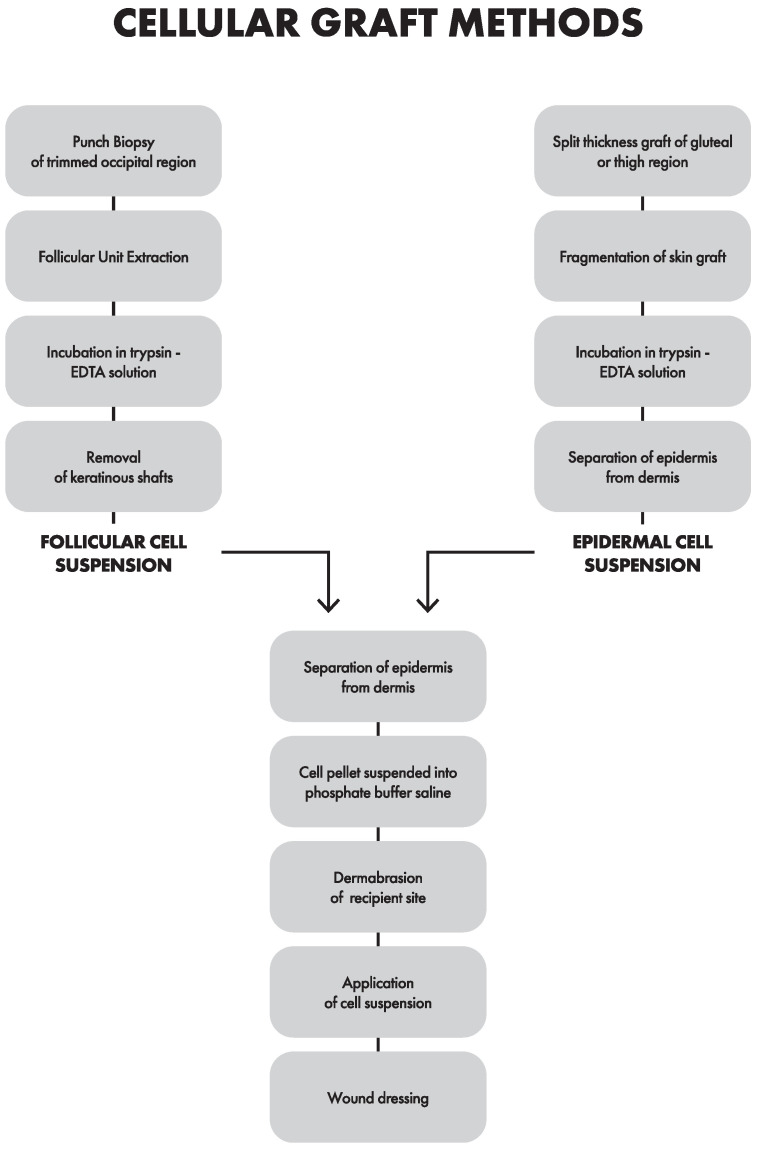
Course of cellular methods.

FCS, also known as noncultured extracted hair follicle outer root sheath cell suspension, was invented in the year 2009 [88]. The idea for transplanting hair follicles came from the observation of a perifollicular repigmentation pattern in some vitiligo patients. This discovery implied the existence of melanocytes and melanocyte stem cells in hair follicles. Both cellular methods share some similarities regarding the preparation of the recipient site and administration of the cell suspension. However, there is a key difference in obtaining the cell suspension (Figure 3). As for material preparation conducted in FCS, the recipient site comes from the occipital region and ranges from 0.5 × 0.5 [89] to 10 × 10 cm [90] in size. The site is trimmed to approximately 2 mm, cleaned with povidone–iodine lotion and, subsequently, anesthetized using 2% lignocaine. A skin punch biopsy of approximately 1 mm is used to obtain follicular units. The punch is rooted in the direction of the hair follicle until it reaches the mid-dermis; yet, caution is advised not to enter subcutaneously. One bundle of hair follicles is obtained from 1 cm^2^ of skin containing approximately 15–25 [75] to, in some cases, 50 pigmented follicles. Subsequently, the follicular units are pulled out using fine forceps with surgical loupes. Sometimes, the follicles are washed three times and then incubated [91]. Then, the acquired hair follicles are collected in a Petri dish containing approximately 4 mL of 0.25% trypsin–EDTA solution. However, Dulbecco’s modified Eagle’s Medium (DMEM) with antibiotics is an alternative for EDTA [91]. Then, there is incubation at 37 °C for 90 min [75,91], and every 30 min, follicles are removed and placed in a new Petri dish containing 0.25% trypsin–EDTA, and the reaction in the previous tube is terminated by adding a trypsin inhibitor. Fetal bovine serum can be used instead as a trypsin inhibitor to prevent the occurrence of cell digestion [75]. Keratinous shafts are discarded, and the acquired cell suspension is then centrifuged for 5 min at 1000 rpm [75,91] or for 10 min at 2500 rpm to obtain a cell pellet, which is located in PBS or DMEM. The quality of the prepared suspension can be measured under a microscope after staining it with trypan blue [90]. The recipient area is thoroughly cleansed and anesthetized, followed by dermabrasion with a motorized dermabrader until pinpoint bleeding is observed. Dermabrasion must be performed in at least two directions [75]. The denuded area is covered with a saline-moistened gauze piece to achieve hemostasis. The previously prepared follicular suspension is aspirated into a 20-gauge needle. It is evenly poured onto the denuded surface and then the recipient area is covered with a collagen dressing, followed by a sterile gauze piece with another dressing. Following the procedure, the patient is allowed to return home, and, on the eighth day, they should present for the dressing removal. In some cases, antibiotics may be prescribed for a week, although this varies by hospital guidelines. After complete re-epithelialization, approximately two weeks [90], patients can be treated with topical tacrolimus or be subjected to phototherapy (PUVA or NB-UVB) at the recipient site until complete/near complete repigmentation occurs or until six months pass. The patient is then scheduled for a follow-up session in the upcoming months. Repigmentation is compared to baseline photographs and transparency sheet tracings during each visit. Adverse effects, if any, should be noted. Overall, hair-follicle-based methods provide more rapid and better repigmentation results, although not statistically significant compared to ECS; nonetheless, it is worth noting that FCS has statistically fewer complications [75]. This method is, overall, the one that requires the greatest effort and has the highest costs. The most common side effects include the infection of the recipient site and color mismatch in the form of hyperpigmentation [91]. As for the modifications, adding collagenase type 1 in addition to trypsin to digest the perifollicular dermal sheath during the preparation of the FCS suspension process could result in increased releases of stem cells from the dermal papilla and dermis. In practice, it does not translate into significantly improved repigmentation [89].

The strengths and limitations of the reported methods are described in Table 2.

## 4. Discussion

Repigmentation is often achieved with surgical methods in the face or trunk areas. However, treating vitiligo requires additional effort when applied to lesions in distal areas or bony prominences, such as the elbows, knees, iliac crest and ankles. The efficacy of treating lesions on bony prominences and the dorsa of hands and feet may depend partly on the nature of melanocyte migration. In methods with diffuse-pattern migration, we observed a better clinical effect than in methods with the repigmentation of a centrifugal type. ECS and SBEG are more effective than MPG in treating lesions on the fingers and toes [55]. Interestingly, although it was not a significant difference, a marginally more rapid and better color-matching effect was produced by SBEG than by ECS. FCS produces slightly better repigmentation, although this is not statistically significant on the head and neck areas compared to acral bony sites [91]. Solutions are being sought that would have a beneficial therapeutic effect on these patients. Among the potential solutions, Muhammed et al. used a combination of two cellular methods, with promising results, obtaining better repigmentation, color-matching and patient satisfaction results than patients treated with epidermal cell suspension alone [78]. There is an issue with the poor effects of the surgical treatment effects of leukotrichia, since none of those methods can replenish lost melanocyte stem cells in the bulge region of the hair follicle. FCS and ECS produce similar poor effects, with percentage repigmentation results in leukotrichia in FCS and ECS groups being 7.42 ± 11.62 and 11.42 ± 17.90, respectively [90].

A technique known as follicular unit extraction (FUE), utilized to treat patches affected by vitiligo with leukotrichia, can provide decent results regarding the cosmetic effect of hair-color matching on lesions [92]. It is assumed that the lighter skin types in the group could provide better chances for a repigmentation effect due to increased melanocytic function and melanization of melanosomes in those skin types [93]. Dermatoscopy is a good way of not only assessing the stability of the disease prior to treatment, but is also an essential tool for monitoring the course of vitiligo treatment and predicting the course of repigmentation (Table 3) [93,94,95,96]. It should be mentioned that leukotrichia, despite the indication of the depletion of the melanocyte reservoir [90], is a favorable prognostic factor for the surgical approach, since it is most commonly associated with the stability of the disease [96]. Wood’s lamp can also be a valuable tool for monitoring patients following surgical procedures. Repigmentation can be recognized as hypochromic islands, which progress to normally pigmented islands [97]. Patients who have some dermatoscopic indications of relapse should be followed up with Wood’s lamp and also provide more accurate surveillance.

The aspect that should be considered when selecting a particular approach to treat vitiligo lesions is size. One of the cellular methods’ most significant advantages is their donor-to-recipient expansion ratio. Thus, 1 cm of skin can repigment 10 cm of skin with ECS. As for MPG, 1 cm can repigment 5 cm, and the SBEG ratio is 1:1 [79]. Cellular methods can cover larger recipient sites than tissue methods [98]; although, even for them, some patients with large lesions treated with ECS may require at least two subsequent procedures [74]. Cellular methods require more appropriate equipment and experienced personnel than tissue methods [75]. As for the healing aspect, the disadvantage of tissue procedures is the slower healing outcome than tissue methods due to melanocytes requiring more time to dissolve than in cell-based methods, where they do so in a diffuse pattern [80]. When comparing cellular methods, the advantages of FCS over ECS are rapid healing outcomes due to the smaller graft size in FCS compared to ECS. However, ECS has been investigated more than FCS in the literature. In theory, FCS should be superior to ECS, considering hair follicles with a higher melanocyte–keratinocyte ratio than the epidermis. A follicular melanin unit constitutes 1 melanocyte per 5 keratinocytes to 1 melanocyte per 36 keratinocytes [92]. In some studies, a higher cellular ratio was achieved in the ECS solution instead of FCS, although it did not contribute to better repigmentation outcomes [90]. However, there is no correlation between the percentage of melanocytes in suspension and clinical repigmentation for FCS [91], although ECS is very reliant on a high donor-to-recipient ratio. When comparing tissue methods, MPG and SBEG have similar efficiency results regarding repigmentation. However, while MPG can be completed within an hour, SBEG usually takes 2–3 h. Moreover, MPG can be performed on sites where SBEG cannot be performed [52]. SBEG had the lowest rate of complications compared to the mini-punch graft [99]. SBEG is very efficient when treating lesions present on the face, neck and limbs [100].

## 5. Conclusions

The effectiveness of the method relies on multiple factors, ranging from a patient’s genetic predisposition for repigmentation to the experience of the facility performing the procedure. Although in recent years, the popularity of cellular methods has essential increased due to their effectiveness in treating extensive lesions, at present, ECS is the tried-true cellular method, as it has been studied more thoroughly in the field; however, given more years, FCS might take that spot. ECS and MPG methods should be prioritized in the treatment of the areas on acral bony sites. Further investigations of modifications that might increase the chances of repigmentation following surgical procedures should be conducted. Dermoscopy is increasingly important in vitiligo-patient assessments as it can accurately estimate the probability of repigmentation prior to and following surgery.

## Figures and Tables

**Table 1:** Predicting factors following epidermal cell suspension.

Higher Rate of Relapse	Lower Rate of Relapse
Male gender	Affected body surface area of less than 1%
Age of surgery older than 23 years	Mechanical dermabrasion
Non-segmental vitiligo	Segmental- or focal-type vitiligo
Age of onset older than 13 years Fingertip involvement	Fingertip involvement Higher rate of repigmentation
New lesion onset	
Laser dermabrasion	
Family history of vitiligo	

**Table 2:** Strengths and limitations of reported methods.

Method	Strengths	Limitations
Suction Blister Epidermal Grafting	⋅ Low cost ⋅ Does not require special equipment ⋅ Efficient for treatment of small lesions, especially on the face, neck and limbs ⋅ It can be applied around the sensitive areas of the mouth and eyelids	⋅ Time-consuming ⋅ May cause slight pain and discomfort to patient ⋅ Slow healing ⋅ Unsuitable for larger areas, uneven surfaces or palms
Mini-Punch Grafting	⋅ Low cost ⋅ Does not require special equipment ⋅ Quick to perform ⋅ Efficient when treating small lesions, especially lips, palms, soles and fingers	⋅ Slow healing ⋅ Risk of cobblestoning (most common complication) ⋅ May be applied to larger lesions; however, requires multiple procedures ⋅ Unsuitable for body folds, oral commissures or non-keratinized mucosa
Epidermal Cell Suspension	⋅ Moderately rapid healing ⋅ Efficient for treatment of large lesions ⋅ Suitable for treatment of areas, such as the elbow, knee, acral, eyelid and mucosa	⋅ High cost ⋅ Time-consuming ⋅ Requires special laboratory equipment
Follicular Cell Suspension	⋅ Fastest healing method ⋅ Efficient for treatment of large lesions ⋅ Fewest number of complications out of all methods ⋅ Suitable for treatment of all areas	⋅ High cost ⋅ Time-consuming ⋅ Require special laboratory equipment ⋅ New method, not as thoroughly investigated as other methods

**Table 3:** Evaluation of dermoscopy.

	Negative Prognostic Factors in Dermoscopy	Positive Prognostic Factors in Dermoscopy
	⋅ Perifollicular pigmentation	⋅ Perifollicular depigmentation
	⋅ Starburst appearance	⋅ Leukotrichia
Prior to treatment	⋅ Koebner phenomenon	⋅ Sharp borders
	⋅ Comet-tail appearance ⋅ Fingertip involvement	
	⋅ Persistence of diffuse pigment specks	⋅ Presence of a normal reticular pigment network
Following treatment	⋅ Altered pigment network	⋅ Marginal and perifollicular hyperpigmentation
	⋅ Telangiectasias	

## Data Availability

Not applicable.
